# Associations between diet and disease activity in ulcerative colitis patients using a novel method of data analysis

**DOI:** 10.1186/1475-2891-4-7

**Published:** 2005-02-10

**Authors:** Elizabeth A Magee, Laurie M Edmond, Shiona M Tasker, San Choon Kong, Richard Curno, John H Cummings

**Affiliations:** 1 Division of Pathology and Neuroscience, Ninewells Hospital and Medical School, Dundee, DD1 9SY, Scotland, UK

**Keywords:** ulcerative colitis, diet, sulfite, thiamin, resistant starch

## Abstract

**Background:**

The relapsing nature and varying geographical prevalence of ulcerative colitis (UC) implicates environmental factors such as diet in its aetiology.

**Methods:**

In order to determine which foods might be related to disease activity in UC a new method of dietary analysis was developed and applied. Eighty-one UC patients were recruited at all stages of the disease process. Following completion of a 7 d diet diary, clinical assessment including a sigmoidoscopic examination (scale 0 (normal mucosa) to 6 (very active disease)) was conducted. Food weights for each person were adjusted (divided) by the person's calorific intake for the week. Each food consumed was given a food sigmoidoscopy score (FSS) calculated by summing the products of the (adjusted) weight of food consumed and sigmoidoscopy score for each patient and occurrence of food and dividing by the total (adjusted) weight of the food consumed by all 81 patients. Thus, foods eaten in large quantities by patients with very active disease have high FSSs and vice versa. Foods consumed by <10 people or weighing <1 kg for the whole group were excluded, leaving 75 foods.

**Results:**

High FSS foods were characterized by high levels of the anti-thiamin additive sulfite (Mann-Whitney, p < 0.001), i.e. bitter, white wine, burgers, soft drinks from concentrates, sausages, lager and red wine. Caffeine also has anti-thiamin properties and decaffeinated coffee was associated with a better clinical state than the caffeine containing version. Beneficial foods (average intake per week) included pork (210 g), breakfast cereals (200 g), lettuce (110 g), apples and pears (390 g), milk (1250 ml), melon (350 g), bananas (350 g), bacon (120 g), beef and beef products (500 g), tomatoes (240 g), soup (700 g), citrus fruits (300 g), fish (290 g), yogurt (410 g), cheese (110 g), potatoes (710 g) and legumes (120 g).

**Conclusions:**

The dietary analysis method described provides a new tool for establishing relationships between diet and disease and indicates a potentially therapeutic diet for UC.

## Background

Ulcerative colitis (UC) is a chronic, relapsing mucosal disorder that extends in continuous fashion proximally from the rectum and is limited to the colon. The aetiology of UC includes a genetic component possibly involving an abnormal cell-mediated immune response to commensal enteric bacteria in the large intestine. The relapse/remission pattern of the disorder and substrate driven nature of microbial metabolism in the large bowel implicate environmental factors such as diet.

Apart from nutritional repletion, dietary measures do not play a role in the management of UC. Nonetheless, attempts to link the cause of UC with specific foods date back at least 50 years[1]. Many foods or food groups have been related to UC (table 1 – see additional file 1) [2-13] including sugar, eggs, soft drinks, fruit and vegetables, protein, carbohydrate and fat. However none have been proven to be of significant benefit or to contribute to the cause of UC. This may partly be because both the assessment of disease activity in UC and dietary intake are difficult to measure, or because the actual dietary component that is key to this relationship has not been measured.

It has been proposed that sulfide, produced in the large bowel from either amino acid fermentation or sulfate reduction, may be a triggering factor in the inflammatory process of UC [14-16]. Recently, in a prospective dietary study where foods rich in sulfur compounds were quantitated, evidence that sulfur compounds may increase the likelihood of subsequent relapse in UC was found[17].

The main source of inorganic sulfur, predominantly sulfate, in the diet are the S (IV) family of additives; the sulfiting agents. Sulfites have been used as food preservatives since the 17th century and are amongst the most widely accepted and versatile of additives. Sulfiting agents, denoted by E220–228 in Europe and generally recognized as safe (GRAS) substances in the USA, include sulfur dioxide, hydrogen sulfites, sulfites and metabisulfites. Sulfiting agents are cheap, easy to use and extremely effective at preventing microbial growth and reducing spoilage[18]. They serve as antioxidants, inhibit enzymatic and non-enzymatic browning reactions and act as a texture modifier in biscuit dough. Sulfites improve color extraction from, and stabilization of grape must in winemaking and preserve lobsters and shrimps from discoloration during iced storage.

However, there are some problems with sulfite use[19,20]. In the early 1980s ingestion or inhalation of sulfites was shown to cause bronchospasm in about 5 % of asthmatics. Sulfite sensitivity can pose a particular threat in the workplace where sulfiting agents are used, but may also occur with ingestion of sulfited foods such as potato products and wine. In addition, skin sensitivity has been reported and there are anti-nutritional effects particularly to thiamin which is readily cleaved by the sulfite ion[21]. The mechanism involves an initial nucleophilic attack to the methylene carbon activated by the positive charge on nitrogen, the reaction rate peaking between pH 5 and 6[18]. As a result of this anti-nutritional effect the GRAS status for sulfites was reviewed in the USA and in 1986 the use of sulfites in fresh and frozen fruit and vegetables revoked and a declaration on the label required[22,23]. Earlier (in the USA) their use in meat had been prohibited, because these foods are an important source of thiamin.

A study of diet and disease activity in UC using a 7 d dietary diary, a full assessment of disease activity and a method of dietary data analysis that allows trends in food consumption not apparent using customary dietary software was therefore undertaken.

## Methods

### Subjects

Eighty-one UC patients were recruited and informed consent obtained. Ethical permission was granted by Tayside Committee on Medical Ethics, Dundee, UK (ref 007/00). As it was important to have a range of disease activities present, recruitment included patients at all stages of the disease. Patients were excluded if clinical examination or histology indicated Crohn's disease or indeterminate colitis, if there was a positive stool culture for pathogens or if the patient had antibiotic treatment within 3 months preceding the start of the study.

### Dietary Assessment

All the UC patients were asked to complete a 7 d diet diary[24]. The diet diary used has been validated for use in the European Prospective Investigation into Cancer study (EPIC). Following completion of the diet diary, subjects attended the research clinic and a full clinical assessment (see below) was carried out. The time interval between the first day of the diary and the clinical visit was on average 28 d. Thus the dietary data is prospective.

7d diet diaries were coded and analyzed using Tinuviel, WISP v3.0 nutritional analysis software (Warrington, UK). Due to the variation in the sulfiting protocols and widespread use of sulfiting agents, current tables of food composition do not contain inorganic sulfur values and cannot be used to quantify intake. Instead of quantitating the intake of particular dietary components, foods and food groups were assessed in their entirety using the method described in the dietary data analysis section (below).

### Clinical Assessment

Clinical assessment included history, physical examination and global clinical grading, plus full blood count, liver function tests and inflammatory markers. Patients were examined by rigid sigmoidoscopy or flexisigmoidoscopy and graded on a scale 0–6 (integers and half integers used) according to the macroscopic appearances of the rectal mucosa at a distance 5–10 cm from the anal verge[25]. The clinical assessment of disease activity was confirmed in each case by histological examination, by a single histopathologist blinded to the clinical details, of a rectal biopsy taken from the posterior rectal wall 5–10 cm from the anal verge[26]. A simple clinical colitis score was assigned to patients on each visit following Walmsley's scoring system[27], together with blood parameters of disease severity (Hb, plasma viscosity, CRP, serum albumin).

### Dietary Data Analysis

Patterns of dietary intake associated with disease activity became apparent through the study of the dietary diaries, e.g. high intakes of sulfite containing foods coupled with a modern processed, convenience diet was associated with a high sigmoidoscopy score. Traditional dietary coding (WISP) did not show any such clear associations between micro or macro nutrient intake and sigmoidoscopy score. Traditional dietary analysis was therefore thought to be missing important patterns in dietary data and a new method of dietary assessment was subsequently developed.

This new method used the following procedure. To calculate the association of a particular food with clinical score, each food or food group consumed was given a food sigmoidoscopy score (FSS) calculated by summing the products of food weight and sigmoidoscopy score for each occurrence of the food or food group and dividing by the total weight of the food or food group contained in all diaries. In order for each diary to make equal contributions to the FSSs, the weight of each food was adjusted using the calorific intake for each person. This procedure was carried out separately for every food item recorded in the 7 d diet diaries but is explained below using the example of red wine.

Red wine score = (Σv(i)s(i))/Σv(i) for i = 1 to 81     equation 1.

Where: -

i is the 7 d dietary diary number (n = 81).

v(i) is the volume (divided by calorific intake for patient (i) of red wine recorded in 7 d dietary diary i.

s(i) is the sigmoidoscopy score associated with 7 d dietary diary (i).

Thus foods eaten in large quantities by patients with high levels of disease activity will have high scores and vice versa. The denominator in the above equation is the total volume of the food in question from all diaries (corrected for calorific intakes) so the food scores can be equated with the effect of a typical portion of the food in question on the sigmoidoscopy scores of the patients. This procedure is repeated for every food item. Foods or food groups were excluded from the analysis if 10 or fewer people consumed them or if they made up less than 1 kg of the total intake of the entire population. The decision as to where food group boundaries lay was made depending on the size of the group and whether the differences between the foods were considered important for this study.

### Statistics and Data Handling

Dietary data was exported from WISP to Microsoft EXCEL 98 (Macintosh version, 1998). A worksheet containing the core headings; Patient ID, food description, weight and patient sigmoidoscopy score was completed. The data was then sorted by food description and each food copied to a separate EXCEL file. Equation 1 was then used to calculate food sigmoidoscopy scores for each food in a manner similar to the example in table 2 (see additional file 2).

Correlation values for scatter plots were obtained using the linear regression function in EXCEL. The equation t = r √((n-2)/(1-r^2^)) combined with t tables provided corresponding significance levels.

## Results

**Figure 1:**
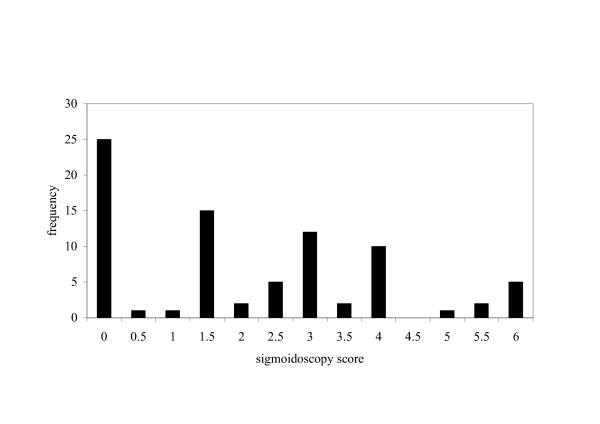
Frequency distribution of sigmoidoscopy scores (n = 81) for ulcerative colitis patients recruited at all stages of disease.

Of the 81 patients recruited 43 were male and 38 female. The average age (range) of the males and females were respectively 53 (26–78) y and 47 (19–74). The distribution of sigmoidoscopy scores is shown in fig 1. One third of the patients had sigmoidoscopy scores of 0, 0.5 or 1. The mean sigmoidoscopy score for all 81 patients was 2.09. The correlation between the clinical activity indexes and sigmoidoscopy scores was r^2 ^= 0.25 (n = 81).

Table 3 (see Additional file 3) shows the foods and food groups with associated sigmoidoscopy scores and average portion sizes. In total 75 foods (or food groups) were given FSS scores. The higher the FSS value the greater the association with disease activity and vice versa. The total weight of foods in all diaries was 1,681 kg. The average food sigmoidoscopy score (i.e. a food sigmoidoscopy score calculated for the entire dietary intake data set was 2.127). Foods excluded from the FSS table (Table 3), by virtue of contributing <1 kg or being consumed by <10 people, made up 8 % of the total weight of all foods and had a score slightly lower (2.001) than that of an average food (2.127). Standard errors are not quoted for the food scores as the data used to generate them (weight * sigmoidoscopy score) was not normally distributed due to the number of sigmoidoscopy scores of 0.

The dietary diaries were assessed for completeness by comparing calorific intakes with expected values for the sexes. Expected (calculated from dietary reference tables using age and sex)[28] versus actual values for men and women were respectively 2481 kcal/d versus 2326 kcal/d and 1925 kcal/d versus 1887 kcal/d.

Foods for which regulations exist in the EU permitting sulfite addition are shown in table 4 (see Additional file 4) [29]. Typically a manufacturer will add sulfite up to the maximum permitted level in order to achieve the longest shelf life for the product. A report on sulfite usage in the UK was produced in 2001[30]. Sweet wines, langoustines (prawns), dehydrated potatoes and dried fruit were not given FSS scores because their data quantity fell below the <10 people or <1 kg rule. Soft drinks were split into those known to contain sulfite (drinks made from fruit squash concentrates and lucozade) and the rest. In terms of intake (portion size*sulfite concentration), for this population, the major sources of sulfite (FSS, FSS table position) were bitter beer (3.91, 75), white wine (2.87, 73), burgers (2.84, 72), soft drink concentrates (2.79, 70), sausages (2.68, 68), lager (2.47, 64) and red wine (2.00, 29). A Mann-Whitney test on the FSS positions of these foods gave a significance of p < 0.001. The sulfite-containing, alcoholic beverages; wines and beers, were associated with increased UC disease activity, but spirits were not, which suggests a role for sulfite rather than alcohol in the disease process. A plot of alcohol consumption from wine and beer against sigmoidoscopy score revealed a significant positive correlation (n = 81, r^2 ^= 0.07, p < 0.02).

Decaffeinated coffee appeared better for the UC patient than the caffeine-containing counterpart. Decaffeinated tea is not shown on table 3 because it was only drunk by 9 people but had a FSS of 1.71 versus 2.01 for the caffeine-containing product. Whole fruit consumption appeared better than the corresponding juice (e.g. fruit juice scored 2.43 compared to citrus fruits at 1.96 and apples at 1.67).

An average thiamin concentration (mg / 100 g) for each food or food group is also shown in table 3. There is a significant correlation (p < 0.005) between this thiamin value and the food's sigmoidoscopy score.

## Discussion

Ulcerative colitis is considered to have a genetic component. Twin studies[31] have shown a 10% concordance of UC in monozygotic and 3% in dizygotic twins suggesting about 90% environmental and 10% genetic contributions. The pool of genetically susceptible individuals is therefore at least 10 times greater than those diagnosed with the condition. A failure to date in identifying the gene(s) responsible points to a complicated genetic component featuring multiple polymorphisms. The first acute episode of UC must disrupt either, the ecology of, or the sensitivity and selectivity of the immune system to, the commensal enteric microflora sufficiently to cause the chronic condition. More extreme versions of the environmental conditions that lead to subsequent relapses could conceivably lead to the first acute episode.

Of all the dietary components studied in relation to UC risk and disease severity, milk has probably received the most attention. Andreson[1] was the first to postulate that food allergy was the cause of UC in two-thirds of his patients, and by the use of elimination diets claimed to identify the offending food and remove it. In Andreson's experience, the most common provoking antigen was cow's milk. His views were confirmed by Rowe[32] and later by Truelove[33]. They all postulated that milk protein sensitivity was an aggravating cause of disease in up to 5% of colitic patients, who benefited from a milk-free diet. While able to demonstrate circulating antibodies to milk proteins more frequently and in higher titer than in matched controls, they were unable to correlate the occurrence and titer of these antibodies with the extent, severity, or duration of colitis, or with the response to a milk-free diet. Mishkin[34] concluded, in a review of the subject, that IBD patients avoid dairy products to a much greater extent than the prevalence of lactose malabsorption and/or milk intolerance in this population group would justify. This observation was probably due to the incorrect perceptions of patients and arbitrary advice of physicians and authors of popular diet books.

In order to ascertain whether dietary antigens may sustain the mucosal inflammatory response, two prospective controlled trials have investigated the effectiveness of bowel rest and total parenteral nutrition as primary therapy in the management of acute UC[35,36]. Neither study found any benefit over conventional corticosteroid treatment alone and so the possibility of a dietary antigen driving the chronicity of the disease seems unlikely. These results are in agreement with work demonstrating[37] that a split ileostomy is of little benefit in the management of UC, but the latter observations may have been confounded by the development of diversion colitis[38].

The dietary analysis procedure proposed here has the potential to highlight trends in dietary data that would not be apparent using traditional dietary analysis software and could be useful in the study of other diseases with dietary associations. This system would highlight any possible dietary factors both positive and negative, not just sulfite. The proposed method is less reductionist than traditional coding as it assesses the risk of each food item or group rather than the risk from the foods' (quantitated) constituents. Part of the power of this study derives from the availability of a sigmoidoscopic grading (0–6) of the severity and extent of the disease. This grading provides the statistical variable that is normally obtained from a non-UC control group. Other alternative systems for analysis of disease risk for dietary components are; the use of disease occurrence odds ratios between the top and bottom quartiles of intakes, and assessing the correlation coefficients between disease activity and intakes. The odds ratio method loses data and data accuracy by characterizing intakes as high, high middle, low middle and low and then discarding the middle two quartiles. The correlation method is dependent on spread. The proposed system has neither of these disadvantages. The food sigmoidoscopy score calculation does rely on the assumption that the sigmoidoscopy score is an approximately linear scale, i.e. a sigmoidoscopy score of 6 is caused by the consumption of a double portion of a harmful food item of sigmoidoscopy score 3. This could be argued to be reasonable. Both the sigmoidoscopy grading and dietary analysis method are validated methodologies. The food sigmoidoscopy score is simply a mathematical function of these two variables. As all data is transformed according to the same simple rules any statistical treatment of the results is as valid as statistical treatment of the raw data.

Whilst clinical activity indices were used to generate analogous scores to the food sigmoidoscopy scores, the results from these measurements are not included in this paper. Clinical activity index involves subjective measurements such as a feeling of well being. Thus, the food orders generated by these measurements were not thought to be as accurate as those generated by the sigmoidoscopy scores.

The consensus of previous studies on diet and UC pointed to the modern, processed, highly refined, Western diets as being damaging. The results presented here linking diet with disease activity are broadly in agreement with this. Additionally they propose a new risk factor for UC, namely intake of sulfited foods.

The involvement of diet in UC is controversial. Differences in dietary intake between patients and controls could be a result of changes in diet brought on by the symptoms of the disease process[4]. While this explanation is possible it does not seem likely that patients would increase their beer and wine intake as a consequence of feeling unwell. The relationship between sulfite intake and sigmoidoscopy score in this study was extremely strong and therefore an explanation for why sulfite should be a risk factor for UC is required. Sulfite has a number of effects that may be relevant to this discussion. Sulfite may be important because it is a precursor of sulfate. Sulfate can potentially be reduced to sulfide by sulfate reducing bacteria in the colon. Sulfide is a plausible metabolic toxin in UC. Supplementing patients with sulfate decreases the microbial incorporation of hydrogen into methane (as measured by breath methane) and increases the in vitro sulfide production rate of feces[39]. The end metabolic product of both sulfite and protein is sulfate. Sulfate from both sources can be reduced to sulfide in the gut. The absence of a significant relationship between protein intake and disease activity in this study does not support a mechanism for UC that involves a common pathway for sulfite and protein.

Alternatively, the relevance of sulfite to UC may be because of its ability to degrade thiamin (particularly at colonic pH). Thiamin deficiency manifests itself in the nervous and cardiovascular systems. It is unlikely that it is the status of the patient that is important, but rather the amount of thiamin available to the gut microflora. An example of the importance of thiamin to the gut microflora is the requirement of the probiotic bacteria, lactobacilli, for thiamin. Thiamin status is influenced by a number of factors. Firstly, thiamin intake; in foods such as pork, fortified cereals and legumes which are good sources of thiamin, intakes were associated with improved clinical state. Traditional dietary analysis did not reveal a significant correlation between thiamin intake and sigmoidoscopy scores though no allowance is made in dietary coding software for the reduction in thiamin content caused by sulfite usage. Secondly, carbohydrate intake; Elmadfa *et al*. demonstrated that the thiamin status of adult humans depends on carbohydrate intake[40]. Carbohydrate (and sugar) intakes have previously been associated with UC relapse (table 1). Finally, thiamin status can be affected by caffeine's anti-thiaminergic properties. For both coffee and tea intake, the decaffeinated version was associated with better clinical state.

However, there was a sub group (n = 8) of this population who recorded an intake of either vitamin B complex or multivitamins. This sub group did not have a mean sigmoidoscopy score significantly lower that the general UC population. It is likely that vitamin B1 is a factor in the disease process but not the only nutritional one.

An additional possible interpretation for the experimentally determined food order is the carbohydrate nature and content of the foods. Carbohydrates, such as the α-amylase resistant starch (RS) and prebiotics, escape digestion in the small intestine and provide an energy substrate for the colonic microflora. Both prebiotics (found in chicory, legumes, artichokes alliums, and in small amounts in cereals) and resistant starch (potatoes, bananas, lentils and legumes) have been hypothesised to improve the colonic health of the host. For RS, resistance to digestion is a function of the morphology of the starch granules and their crystalline organisation, which is determined by the botanical source of the starch and the processing it has undergone before being eaten[41]. Prebiotics are non-digestible carbohydrates that selectively stimulate the growth of lactobacilli and bifidobacteria with benefit to health. Prebiotics are mainly fructose and galactose polymers with a degree polymerisation of between 2 and 60. Of the prebiotic sources; chicory and artichokes were not found in typical diets, legumes and cereals were seen to have probable benefits in this study and alliums were not. This study therefore provides only limited support for the use of prebiotics in UC. The foods containing RS were all found to be of benefit in this study and therefore the role of RS in UC is strongly supported.

Any dietary advice provided to ulcerative colitis patients should be based on the FSS table. The table is of course imperfect because of experimental error, natural variation and the associations between foods. For example, milk and cereal are coded separately but are often consumed together. Thus the magnitude of the difference in the FSSs for these two foods is less than if they'd been independent variables. Suggestions have been made in this discussion as to the factors responsible for the FSS order and to distill these factors into the advice given in Table 5 (see Additional file 5). This table is speculation, as this diet has not been formally tested in the UC population. It does however represent the only comprehensive dietary advice available to ulcerative colitis patients at this time.

The list of dietary risk factors for colon cancer[42] bears a similarity to the dietary risk factors presented here for UC. UC patients have an increased risk of colorectal cancer and it is probable that factors responsible for inflammation in UC patients are also responsible for neoplasia in the colon cancer population.

## Conclusion

A dietary analysis method is described that provides a new tool for establishing relationships between diet and disease. This method has been applied to the study of ulcerative colitis and points to sulfite and caffeine as being harmful, with thiamin and resistant starch being potentially therapeutic. For the first time, dietary guidelines for ulcerative colitis patients, including food portion sizes have been developed.

## Abbreviations

Ulcerative colitis (UC); Food sigmoidoscopy score (FSS); European Union (EU); Odds ratio (OD) and Confidence interval (CI).

## Competing interests

The author(s) declare that they have no competing interests.

## Authors' contributions

JHC, EAM and LME contributed to the study design and the writing of the manuscript. EAM was the co-ordinator of the study and along with ST had responsibility for managing the patients and coding the diaries. LME developed the dietary data analysis protocol assisted by RC. CK and JHC performed the sigmoidoscopic examinations of the patients. All authors read and approved the final manuscript.

## Supplementary Material

Additional File 1 Review of studies of diet and ulcerative colitis (UC). Click here for file

Additional File 2 Food sigmoidoscopy score (FSS) calculation example for red wine (NB incomplete data set used). Click here for file

Additional File 3 Foods consumed in order of food sigmoidoscopy scores (FSS)[43]. Click here for file

Additional File 4 Permitted levels of sulfite in the UK. Click here for file

Additional File 5 Proposed dietary advice for ulcerative colitis patients[44]. Click here for file
