# Supplementary material for: Associations between diet and disease activity in ulcerative colitis patients using a novel method of data analysis
Source: Nutr J. 2005 Feb 10;4:7. doi: 10.1186/1475-2891-4-7 (PMC549081; doi:10.1186/1475-2891-4-7)
Supplement: Additional File 1 — Review of studies of diet and ulcerative colitis (UC). [file 1475-2891-4-7-S1.doc]

Table 1: Review of studies of diet and ulcerative colitis (UC).

| **Food group(s)** | **Association with UC** | **Number** | **Comment (s)** | **Author (s)** |
| --- | --- | --- | --- | --- |
| Sugar | Positive  Positive  Positive  Positive | 100 UC, 100 controls  124 UC, 250 controls  54 UC, 108 matched controls  20 UC | NS  OR 2.37  p=0.05  NS | Mayberry *et al* 1980 ^2^  Porro & Panza 1985 ^3^  Reif *et al* 1997 ^4^  Hart *et al* 2002 ^5^ |
| Carbohydrate | Positive  Positive | 20 UC  53 UC, 106 controls | NS  p<0.001 | Hart *et al* 2002 ^5^  Tragnone *et al* 1995 ^6^ |
| Fibre | No benefit | 39 UC | 6 months high fibre diet | Davies & Rhodes 1978 ^7^ |
| Breakfast cereals | No benefit  Negative | 61 UC, 61 matched controls  114 UC, 114 controls | NS  p<0.05 | Archer & Harveys 1978 ^8^  Brandes *et al* 1979 ^9^ |
| Fruit, fruit juice and vegetables | Negative  Negative  Negative  Negative | 114 UC, 114 controls  197 UC, 394 matched controls  54 UC, 108 matched controls  124 UC patients, 250 controls | p<0.05  OR 0.77 (CI 0.45-1.35)  Fruit OR p=0.36  Veg OR p=0.57  Fruit OR 0.3, Veg OR 0.38 | Brandes *et al* 1979 ^9^  Gilat *et al* 1981 ^10^  Reif *et al* 1997 ^4^  Porro and Panza 1985 ^3^ |
| Eggs | Positive | 54 UC, 108 matched controls | p=0.05 | Reif *et al* 1997 ^4^ |
| Protein | Positive  Positive | 53 UC, 106 controls  54 UC, 54 controls | p<0.001  p=0.65 | Tragnone *et al* 1995 ^6^  Reif *et al* 1997 ^4^ |
| Margarine | No effect  Positive | 80 UC, 73 controls  101 UC, 143 controls | NS  p=0.005 | Chuah *et al* 1992 ^11^  Kono *et al* 1994 ^12^ |
| Fat | Positive | 20 UC | NS | Hart *et al* 2002 ^5^ |
| Western foods | Positive | 101 UC, 143 controls | p=0.04 | Kono *et al* 1994 ^12^ |
| Soft drinks | Positive | 54 UC, 108 matched controls | p=0.02 | Reif *et al* 1997 ^4^ |
| Fish | Negative  Negative | 54 UC, matched controls  20 UC | p=0.36  NS | Reif *et al* 1997 ^4^  Hart *et al* 2002 ^5^ |
| Fast foods | Positive | 145 UC, 305 matched controls | OR 3.9 95% CI (1.4-10.6) | Persson *et al* 1992 ^13^ |
