# Supplementary material for: Associations between diet and disease activity in ulcerative colitis patients using a novel method of data analysis
Source: Nutr J. 2005 Feb 10;4:7. doi: 10.1186/1475-2891-4-7 (PMC549081; doi:10.1186/1475-2891-4-7)
Supplement: Additional File 2 — Food sigmoidoscopy score (FSS) calculation example for red wine (NB incomplete data set used). [file 1475-2891-4-7-S2.doc]

Table 2: Food sigmoidoscopy score (FSS) calculation example for red wine (NB incomplete data set used). The final column value for patient 3 is 3.5x750/2087=1.258. The values in the final column are summed and in this example the food sigmoidoscopy score for red wine calculated as 2.614/0.883=2.96.

|  | **Patient ID** | **Food description** | **Weight ml** | **Diary energy kcal/day** | **Weight / diary energy** | **Sigmoidoscopy score** | **Sigmoidoscopy score * amount / diary energy** |
| --- | --- | --- | --- | --- | --- | --- | --- |
|  | 3  4  23  28 | Red wine  Red wine  Red wine  Red wine | 750  188  525  375 | 2087  1800  2159  2132 | 0.359  0.104  0.243  0.176 | 3.5  6  3  0 | 1.258  0.627  0.730  0.000 |
| **sum** |  |  | **1838** |  | **0.883** |  | **2.614** |
