# Supplementary material for: Associations between diet and disease activity in ulcerative colitis patients using a novel method of data analysis
Source: Nutr J. 2005 Feb 10;4:7. doi: 10.1186/1475-2891-4-7 (PMC549081; doi:10.1186/1475-2891-4-7)
Supplement: Additional File 3 — Foods consumed in order of food sigmoidoscopy scores (FSS)[43]. [file 1475-2891-4-7-S3.doc]

Table 3: Foods consumed in order of food sigmoidoscopy scores (FSS). No foods contribute to more that one category (with the exception of the field "all foods"). The thiamin levels are the best estimate based on the distribution of foods within each group.

| **FSS**  **order** | **food** | **FSS**  **score** | **n** | **total**  **weight g** | **average portion**  **per week / g** | **thiamin ^43^**  **mg/100g** |
| --- | --- | --- | --- | --- | --- | --- |
| 1 | crackers | 1.003 | 22 | 1465 | 67 | 0.24 |
| 2 | pork | 1.219 | 35 | 7511 | 215 | 0.61 |
| 3 | coffee (decaffeinated) | 1.24 | 15 | 30298 | 2020 | Trace-0.04 |
| 4 | corn flakes | 1.451 | 14 | 1557 | 111 | 1.2 |
| 5 | breakfast cereals (not cornflakes) | 1.465 | 46 | 8866 | 193 | 1.16 |
| 6 | lettuce | 1.553 | 48 | 5427 | 113 | 0.12 |
| 7 | butter | 1.557 | 48 | 3795 | 79 | Trace |
| 8 | pizza | 1.6 | 25 | 5599 | 224 | 0.2 |
| 9 | custard | 1.624 | 16 | 3034 | 190 | 0.09 |
| 10 | apples, raw | 1.668 | 42 | 13508 | 322 | 0.03 |
| 11 | sweetcorn | 1.747 | 22 | 1852 | 84 | 0.07 |
| 12 | spirits | 1.776 | 32 | 6339 | 198 | 0.00 |
| 13 | milk, all types | 1.807 | 74 | 92733 | 1253 | 0.04 |
| 14 | melon | 1.816 | 14 | 4846 | 346 | 0.03 |
| 15 | pears, raw | 1.82 | 19 | 5261 | 277 | 0.02 |
| 16 | cucumber | 1.854 | 26 | 1259 | 48 | 0.03 |
| 17 | ice cream | 1.856 | 36 | 4666 | 130 | 0.08 |
| 18 | bananas | 1.892 | 58 | 20400 | 352 | 0.04 |
| 19 | bacon | 1.901 | 50 | 5925 | 119 | 1.03 |
| 20 | beef (and products) | 1.905 | 64 | 32009 | 500 | 0.08 |
| 21 | tomatoes | 1.912 | 60 | 14634 | 244 | 0.09 |
| 22 | jam and marmalade | 1.941 | 45 | 2984 | 66 | Trace |
| 23 | chocolate (and products) | 1.946 | 74 | 17200 | 232 | 0.1 |
| 24 | sugar and sweets | 1.95 | 58 | 5409 | 93 | 0.0 |
| 25 | soup | 1.953 | 55 | 38829 | 706 | 0.05 |
| 26 | Miscellaneous wheat flour products | 1.957 | 43 | 9382 | 218 | 0.21 |
| 27 | citrus fruits | 1.961 | 35 | 10309 | 295 | 0.09 |
| 28 | mayonnaise | 1.985 | 23 | 2375 | 103 | 0-0.02 |
| 29 | red wine | 2.000 | 20 | 10082 | 504 | Trace |
| 30 | tea (caffeine) | 2.008 | 64 | 258121 | 4033 | Trace |
| 31 | fish | 2.038 | 59 | 16869 | 286 | 0.08 |
| 32 | yogurt | 2.049 | 44 | 18215 | 414 | 0.1 |
| 33 | cheese | 2.074 | 61 | 6779 | 111 | 0.04 |
| 34 | mushroom | 2.076 | 21 | 1389 | 66 | 0.09 |
| 35 | potatoes (new and old) | 2.077 | 74 | 38039 | 514 | 0.2 |
| 36 | peas and beans | 2.082 | 56 | 6669 | 119 | 0.16 |
| 37 | chips | 2.102 | 56 | 16286 | 291 | 0.12 |
| 38 | soft drinks (not from concentrate) | 2.104 | 53 | 72169 | 1362 | 0-Trace |
| 39 | white bread | 2.107 | 78 | 36673 | 470 | 0.22 |
|  | all foods | 2.127 | 81 | 1680750 | 20750 |  |
| 40 | turkey | 2.131 | 16 | 2786 | 174 | 0.07 |
| 41 | ham | 2.132 | 48 | 5088 | 106 | 0.58 |
| 42 | crisps | 2.141 | 49 | 4312 | 88 | 0.21 |
| 43 | eggs | 2.158 | 65 | 10397 | 160 | 0.07 |
| 44 | biscuits | 2.162 | 55 | 4613 | 84 | 0.15 |
| 45 | margarine | 2.198 | 56 | 5071 | 91 | Trace |
| 46 | cakes | 2.209 | 62 | 12342 | 199 | 0.13 |
| 47 | cream | 2.215 | 17 | 1677 | 99 | 0.03 |
| 48 | cranberry | 2.224 | 10 | 3549 | 355 | Trace |
| 49 | vegetables (miscellaneous) | 2.235 | 32 | 6635 | 207 | 0.1 |
| 50 | gravy (instant) | 2.242 | 25 | 1892 | 76 | 0 |
| 51 | coffee (caffeine) | 2.247 | 57 | 130423 | 2288 | Trace-0.04 |
| 52 | porridge | 2.269 | 17 | 7411 | 436 | 0.08 |
| 53 | peppers | 2.28 | 20 | 2067 | 103 | 0.01 |
| 54 | baked beans | 2.311 | 30 | 5371 | 179 | 0.09 |
| 55 | rice | 2.315 | 47 | 15614 | 332 | 0.04 |
| 56 | strawberry and raspberry | 2.324 | 11 | 1195 | 109 | 0.03 |
| 57 | brown bread | 2.356 | 50 | 15910 | 318 | 0.26 |
| 58 | chicken (and products) | 2.358 | 69 | 31208 | 452 | 0.07 |
| 59 | fruit pies | 2.362 | 26 | 3909 | 150 | 0.08 |
| 60 | pasta | 2.377 | 45 | 16519 | 367 | 0.03 |
| 61 | peaches | 2.396 | 10 | 2460 | 246 | 0.02 |
| 62 | fruit juice (pure) | 2.429 | 45 | 32904 | 731 | 0.06 |
| 63 | onions (including leeks) | 2.466 | 37 | 2351 | 64 | 0.07 |
| 64 | lager | 2.468 | 16 | 24111 | 1507 | Trace |
| 65 | brassicas | 2.562 | 55 | 10069 | 183 | 0.07 |
| 66 | lamb | 2.582 | 12 | 2205 | 184 | 0.12 |
| 67 | carrots | 2.653 | 47 | 5010 | 107 | 0.08 |
| 68 | sausages | 2.679 | 37 | 7184 | 194 | 0.01 |
| 69 | grapes | 2.713 | 22 | 3744 | 170 | 0.05 |
| 70 | soft drinks (from concentrate) | 2.794 | 31 | 37279 | 1203 | Trace-0.02 |
| 71 | cereal bars | 2.831 | 14 | 1253 | 90 | 0.24 |
| 72 | burgers | 2.838 | 10 | 1125 | 113 | 0.07 |
| 73 | white wine | 2.868 | 16 | 11951 | 747 | Trace |
| 74 | kiwi fruit | 3.471 | 13 | 1474 | 113 | 0.01 |
| 75 | bitter | 3.906 | 10 | 51408 | 5141 | Trace |
