# Supplementary material for: Associations between diet and disease activity in ulcerative colitis patients using a novel method of data analysis
Source: Nutr J. 2005 Feb 10;4:7. doi: 10.1186/1475-2891-4-7 (PMC549081; doi:10.1186/1475-2891-4-7)
Supplement: Additional File 4 — Permitted levels of sulfite in the UK. [file 1475-2891-4-7-S4.doc]

Table 4: Permitted levels of sulfite in the UK.

ProductPermitted sulfite level ^29^beer20 mg/kg (50 mg/kg 2nd fermentation)white wines210 mg/Lred wines 160 mg/Lsweet wines 300-400 mg/Lbreakfast sausages450 mg/kgburgers450 mg/kgsoft drink concentrates20 mg/l, 250 mg/L or 350 mg/Ldried fruit50 (dried coconut)- 2000 mg/kgdehydrated potatoes 400 mg/kgcarbonated drinks20 mg/L (carry over from concentrates only)langoustines150 mg/kg edible part (raw)jams and marmalade50 mg/Ldry biscuits50 mg/kgfrozen potatoes100 mg/kgpeeled potatoes50 mg/kgjams, jellies and marmalades made with sulfited fruit100 mg/L
