# Supplementary material for: Associations between diet and disease activity in ulcerative colitis patients using a novel method of data analysis
Source: Nutr J. 2005 Feb 10;4:7. doi: 10.1186/1475-2891-4-7 (PMC549081; doi:10.1186/1475-2891-4-7)
Supplement: Additional File 5 — Proposed dietary advice for ulcerative colitis patients[44]. [file 1475-2891-4-7-S5.doc]

Table 5: Proposed dietary advice for ulcerative colitis patients. The list of potentially protective foods includes the average 7 d intakes for foods. The total calorific content of the protective foods is approximately 30 % of that required for a whole week. Grams (g) can be converted to ounces by dividing by 28.4. It is recommended that the majority of the protective foods be consumed in the quantities listed. Provided sulfite containing foods and coffee (except decaffeinated) are restricted all other foods can be eaten freely. A varied, fresh, balanced diet is recommended.

*Carrageenan is to be avoided because of its link to ulcerative colitis in animal models[44]. It is a type of seaweed that contains sulphur (used as a thickening agent in a wide range of desserts, e.g. chocolate mousse). This study was not able to determine whether or not this additive is harmful so advice is precautionary.

| **POTENTIALLY PROTECTIVE DIET (weight per week)** | **FOODS TO AVOID OR REDUCE** |
| --- | --- |
| - **pork (210 g)** - **breakfast cereals (200 g), with milk** - **lettuce (110 g)** - **apples or pears, raw (390 g)** - **melon (350 g)** - **bananas (350 g)** - **bacon (120 g)** - **beef (250 g), or beef products (500 g)** - **tomatoes (240 g)** - **soup (700 g), not dried** - **citrus fruits (300 g)** - **fish (290 g)** - **yogurt (410 g)** - **cheese (110 g)** - **potatoes, including chips (710 g)** - **legumes (peas and beans) (120 g)** | - **Beer**   **(except German beer which is sulphite free)**   - **Wine (particularly white)** - **Sausages and burgers**   **(unless not sulphited, e.g. some organic brands)**   - **Sulfite containing soft drinks**   **(e.g. fruit squash concentrates)**   - **Prawns, scampi and shellfish**   **(usually sulfited)**   - **Sulfited dried fruit and vegetables**   **(e.g. dried potatoes, dried apricots)**   - **Processed fruit pies and fruit cakes** - **Coffee (except decaffeinated)** - **Any other foods containing the sulfite additives E220-228** - **Any foods containing carrageenan (Irish moss), E407*.** |
